# A temporal precedence based clustering method for gene expression microarray data

**DOI:** 10.1186/1471-2105-11-68

**Published:** 2010-01-30

**Authors:** Ritesh Krishna, Chang-Tsun Li, Vicky Buchanan-Wollaston

**Affiliations:** 1 Department of Computer Science, Warwick University, Coventry CV4 7AL, UK; 2 Warwick Horticulture Research Institute, University of Warwick, Wellesbourne CV35 9EF, UK

## Abstract

**Background:**

Time-course microarray experiments can produce useful data which can help in understanding the underlying dynamics of the system. Clustering is an important stage in microarray data analysis where the data is grouped together according to certain characteristics. The majority of clustering techniques are based on distance or visual similarity measures which may not be suitable for clustering of temporal microarray data where the sequential nature of time is important. We present a Granger causality based technique to cluster temporal microarray gene expression data, which measures the interdependence between two time-series by statistically testing if one time-series can be used for forecasting the other time-series or not.

**Results:**

A gene-association matrix is constructed by testing temporal relationships between pairs of genes using the Granger causality test. The association matrix is further analyzed using a graph-theoretic technique to detect highly connected components representing interesting biological modules. We test our approach on synthesized datasets and real biological datasets obtained for Arabidopsis thaliana. We show the effectiveness of our approach by analyzing the results using the existing biological literature. We also report interesting structural properties of the association network commonly desired in any biological system.

**Conclusions:**

Our experiments on synthesized and real microarray datasets show that our approach produces encouraging results. The method is simple in implementation and is statistically traceable at each step. The method can produce sets of functionally related genes which can be further used for reverse-engineering of gene circuits.

## Background

Microarrays allow simultaneous measurement of thousands of genes in a short span of time. This approach provides abundant opportunities for scientists to detect and experimentally validate the hypothesis that the data might be generating. Microarray experiments have traditionally focused on measurement of gene expressions at a single time point and are increasingly being applied to measure expression-levels across multiple time points. Such time-course measurements can help in gaining insights into the dynamics of gene interactions [1-3]. The computational analysis of temporal microarray data requires three distinct stages to be performed before some meaningful hypothesis from data can be derived. The first stage is the normalization stage where data is cleaned from the effects of unwanted experimental biases [4,5]. The second stage requires the grouping of data based on certain features which helps in reduction of data dimensions. The third and final stage is the inference of relationship between various genes of interest and understanding the functioning of smaller subsystems which comprise together to make a bigger system. Though these three stages have an ordered sequence of execution, the computational methods applied at these stages need not be dependent on each other. The normalization method solely relies on the experimental design of the microarray experiment [5,6]. The clustering step can be performed using point-based, model-based or feature based grouping of data [7] depending on the hypothesis adopted by the practitioner. The final stage of relationship inference between genes is restricted to the sets of *selected *genes which can be studied as a system of bivariate or multivariate causal *interactions*. Keeping in mind, the final goal of microarray data analysis being identification of *interactions *between genes at the third level, the quest for this goal should ideally start when the data is being grouped together at the clustering stage. One of the ultimate goals of all gene clustering algorithms is to discover the underlying gene pathways representing the biological processes. Genes that are lying in the same pathway are often activated or depressed *simultaneously *or *sequentially *upon receiving stimuli. The biological signal is typically transmitted through intermediate gene interactions due to physical or chemical activities. The simultaneous or sequential activation, or depression, is delineated by the underlying network connection patterns. In this paper, we present a novel approach for clustering of temporal microarray data based on the notion of temporal interaction between the genes. The temporal recording of gene expressions provides an excellent opportunity to view the gene profiles with respect to time and helps in understanding the underlying causal processes driving the behavior of the genes and the system in turn. Like any dynamical system, in a system with a temporal expression profile, time plays a crucial role in the way the system behaves. The primary hypothesis behind the approach presented in this paper is: *the observed effect on any gene is due to some cause propagated over time*. The observed expression of a gene could be due to the effect of other genes present in the system which may be activating or inhibiting the gene under observation with different time-lags. In other words, *we perceive the system as a set of interacting entities, where each entity is a stochastic process and the interactions between them are temporal activities taking place between a pair of processes*.

A system with such behavior is a widely accepted concept in Economics and Neuroscience. Granger [8] proposed a method to evaluate the influence of one time series on the other time series. Granger causality has recently been introduced in bioinformatics [9-12] to reverse-engineer gene circuits from microarray data. We will utilize Granger causality in conjunction with a graph-theoretic method to build an association matrix for the genes and detect the functional modules present in the data. A *functional module *can be defined as a separate substructure of a network having a group of genes or their products that are related by physical or genetic interactions. In graph-theoretic sense, a functional module can be represented by highly connected regions in a network, where the functions are predicted using connections in a graph based on the assumption that genes which lie close to one another are more likely to have similar functions or constitute gene complexes [13,14]. We will also analyze that how the association network obtained by us has certain architectural properties desired from biological networks [15] which distinguishes it from a randomly generated network.

### Related Work

There are many clustering techniques proposed for clustering of gene expression data. However, majority of these techniques do not take into account the sequential nature of time series data, and thus are inappropriate for clustering such datasets. The earlier proposed approaches can broadly be divided into three categories.

1. Point-wise distance based methods - group genes by minimizing an objective function based on a distance measure computed between gene pairs. The distance measure could be Euclidean distance, mutual information, correlation or its respective variants [16], etc. The point-wise methods can be further classified into two classes: (a) partitioning, and (b) hierarchical. Among partitioning methods, k-means [17] and self-organizing maps (SOM) [18] are widely used approaches. Hierarchical methods on the other hand create a hierarchy of relative distances and place multinomial points along a one-dimensional axis based on the relative distance between points. A typical representation of results obtained from hierarchy based methods is in the form of a dendrogram [19]. Point-wise distance based approaches are the most widely used clustering techniques for gene expression data due to their computational and conceptual simplicity. These methods are also popular due to their implementation in the large number of software packages designed for analysis of gene expression data. Some biological case studies using point-wise methods for clustering gene expression data can be found in [20-22].

2. Feature based clustering methods - aim at detecting salient features and local or global shape characteristics of the expression profiles. As opposed to a distance based similarity measure, looking for general shape among the gene profiles can uncover more intricate relationships, such as time shifts and inversion in expression profiles. Ji and Tan [23] proposed a time-lagged based cluster identification technique which relies on the directional change of profiles across consecutive time points. Edge detection method by Chen et al. [24] sums the number of edges of two gene expression curves where edges have the same direction within a time lag to generate a score. Directional changes were also used to compute the slope of expression values in Event method by Kwon et al. [25] to cluster the gene profiles. Some of the feature based clustering methods transform the raw expression data to symbols which are further analyzed to detect similarity between profiles [26,27]. Dominant Spectral Component Method by Yeung et al. [28] decomposes temporal expression sequences into spectral components using the autoregressive modeling technique to measure gene-gene relationship to form clusters. Graph-theoretic approaches studying the nature, properties, structure of the graph where the genes represent the nodes and the arcs representing association between genes also come under feature based clustering methods. Graph spectral clustering [29] and minimal spanning tree methods [30,31] are other well-known feature based clustering methods.

3. Model based clustering methods - shift the similarity emphasis from the data to the unknown model that describes the data. Such methods are based on statistical mixture models which assume that data is generated by a finite mixture of underlying probability distributions, with each component corresponding to a distinct cluster [32-35]. Model based clustering relies on the fundamental assumption that the observed expression profiles are clustered in functional space based on their characteristics. The focus of this approach is in functional decomposition of data, rather than the decomposition of raw data. The computational approach in model based clustering methods is based on maximizing the likelihood of data points. Expectation-maximization (EM) is a popular model based clustering approach to estimate unknown parameters (mean and standard deviation in case of Gaussian distribution) of underlying probability distribution for each cluster in order to maximize the likelihood of the observed expression profiles [36]. Based on similar lines as EM algorithm, Schliep et al. [37,38] suggested gene clustering based on a mixture of Hidden Markov Model (HMM). Along the similar thoughts that a time-course gene dataset is a set of time series generated by stochastic processes, Ramoni et al. [39] suggested the use of autoregressive representation for each stochastic process defining a cluster. This method relies on regression and groups together genes whose dynamics can be expressed with roughly the same auto-regressive equation. Bar-Joseph et al. [40] presented a clustering algorithm that uses splines to cluster the continuous representation of time series expression data. In some cases, prior knowledge has been used to fit the models to the expression profiles. For example, Zhao et al. [41] and Lu et al. [42] have used sinusoids to identify yeast genes with cyclic behaviour. Moller-Levet et al. [43] presented a method based on a predefined comprehensive set of profiles to cluster genes according to their match with respective profiles.

The method proposed in this paper for clustering of temporal gene expression data takes advantage of the essential behaviour of the Granger causality test, which determines if one time-series is useful in forecasting the other time-series or not. The network obtained after applying the Granger causality test is representative of the association between gene-pairs which pass the test. In order to detect the potential functionally related genes, we use a graph theoretical technique to detect dense regions in the association network. Our approach shows that the detection of dense regions in association with Granger causality test plays an equally important role in the proposed clustering technique. The method is tested using both synthetic as well as real datasets obtained to monitor senescence in Arabidopsis Thaliana. To the best of our knowledge, this is a new approach in clustering of temporal gene-expression data which can be used for automated grouping of interesting genes from a large dataset.

## Results and Discussion

### Experiments with Synthetic Datasets

We test our method on three sets of synthetic multivariate datasets. Each set represents a collection of stochastic processes in the form of time-series. We construct each set in such a way that the processes belonging to the set are interdependent, whereas the sets themselves are disjoint from each other.

*Dataset 1*:

*Dataset 2*:

*Dataset 3*:

In the above datasets, ϵ_*i *_~ N(0,1) represents the uncorrelated random error associated with each process. In Dataset 1, *x*_1 _is the driving force for *x*_2_, *x*_3 _and *x*_4 _with time lags 2,3 and 2 respectively. *x*_4 _further drives *x*_5 _and they both share a feedback loop. Similarly, in Dataset 2, *x*_1 _drives *x*_2 _with time lag 3 and *x*_2 _in turn drives *x*_3_. *x*_1 _and *x*_3 _both together drive *x*_4_. Similarly, in Dataset 3, we have *x*_1 _driving *x*_2_. *x*_2 _drives *x*_3 _with lag 2 and *x*_3 _in turn drives *x*_4_. The process *x*_5 _is driven by *x*_2 _and *x*_4 _with time lag 2 and 1 respectively. In the end, *x*_6 _receives the drives from *x*_1_, *x*_5 _and *x*_3 _with time lags 2,1 and 3 respectively.

The datasets are disjoint from each other due to different sources of initiation. The datasets show different arrangements of connections between the processes which include feedback loops, low and high coefficients of drive between processes, multiple processes together driving a single process and all the processes interacting with other processes on a different time lag.

**Figure 1:**
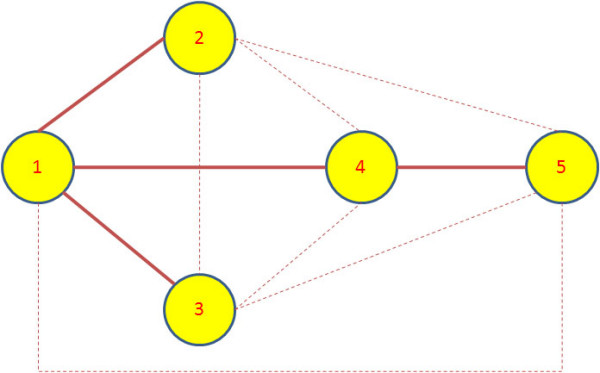
**Inferred network for Dataset 1**. The network structure inferred after applying Granger causality test on the synthetic dataset 1.

**Figure 2:**
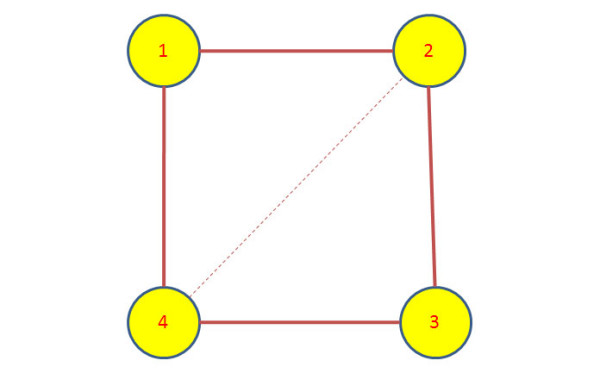
**Inferred network for Dataset 2**. The network structure inferred after applying Granger causality test on the synthetic dataset 2.

**Figure 3:**
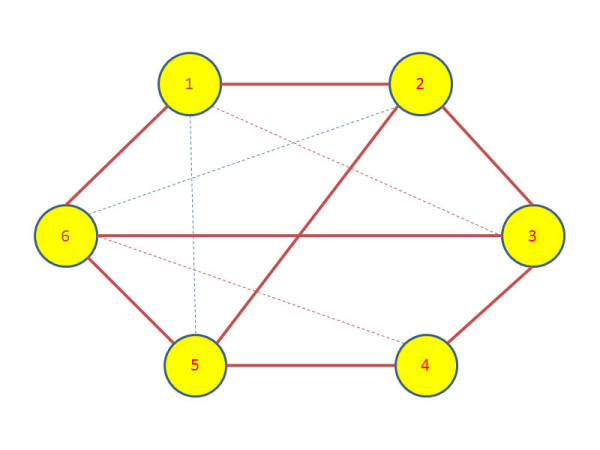
**Inferred network for Dataset 3**. The network structure inferred after applying Granger causality test on the synthetic dataset 3.

We apply the Granger causality to infer the interactions between different entities in each dataset. The Granger causality test was implemented in Matlab and the source code is available on request from the first author. The standard critical value of *α *= 0.05 was chosen for the F-test to accept or reject the hypothesis. The causal hypothesis *H*_0 _was tested for each pair of processes denoted by (*X*, *Y*) in both ways i.e, *X *causing *Y*, and *Y *causing *X*. Since we are only interested in the presence of interaction between (*X*, *Y*), we ignore the directionality of causal influence and quantify the association between the pair with the higher of causality value obtained from both directions. If there is no causal relationship between the pair, the association between *X *and *Y *is quantified as zero. The networks obtained after computing the Granger causality and weighing the edges for all the synthetic datasets are shown in Figures 1, 2 and 3. The true edges according to the equations describing the datasets are plotted with solid bold lines, whereas the extra detected edges are plotted with thin dashed lines.

We see in Figure 1 for (Dataset 1) that node 1 connects to nodes 2,3 and 4. Nodes 4 and 5 are also connected in the inferred network structure. The equations describing the Dataset 1 reflect these facts. One of the extra link present is the interaction of node 2 with node 3 showing the fact that nodes 2 and 3 are both driven by node 1. They exhibit an interaction according to the F-test criteria but their strength is very low compared to other interactions. Since node 1 is also a driving force for node 4, so according to the previous argument, nodes 2 and 3 are also found to drive node 4. Node 4 and node 5 share a feedback loop, thus an interaction between them exists. There is a similar situation with nodes 1,2 and 3 interacting with node 5 due to node 1 being the common driving force behind nodes 2 and 3.

The connections are simpler and more sparse in the case of Figure 2 for Dataset 2 where there is an extra edge not described by the system of equations is present in the inferred network. Similarly in the network obtained for Dataset 3, the influence of node 1 on nodes 3 and 5 can be attributed to the fact that the influence is propagating through node 2 which is directly regulating nodes 3 and 5. The influence of node 2 on 6 is due to node 2 being the driving force for node 3 which in turn is directly influencing node 6. In the similar fashion, the dashed line between nodes 4 and 6 can be explained due to node 4 driving node 5 which in turn is driving node 6.

**Figure 4:**
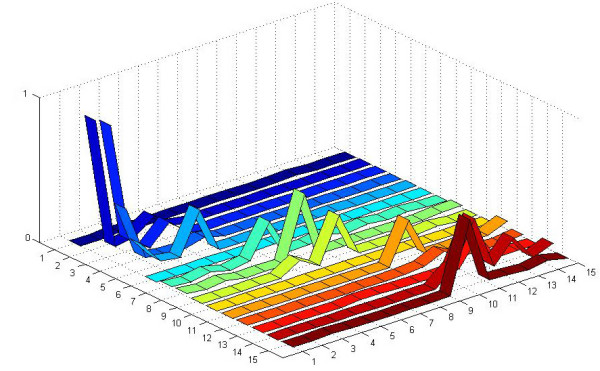
**Simulation results with Dataset 1, 2 and 3 integrated into one system**. The association graph obtained after applying the Granger causality test on the combined dataset is represented in form of a association matrix. We can see three distinct island like modules in the graph, each module representing a dataset.

Having analyzed the individual datasets, we further investigate what happens when all the three datasets are put together to form a bigger system of processes and the pairwise interaction between the processes are computed. We create a system of 15 entities where the first 5 entities represented the processes in Dataset 1, the entities from 6 to 9 represented the processes from Dataset 2, and the last 6 entities represented the processes in Dataset 3. We then test for Granger causality for all possible pairs of processes (total 210 directional edges for a complete network with 15 nodes) in the system. We plot the interaction strength between the processes in Figure 4 where the *x *and *y *axes represent the 15 × 15 matrix of processes in the system. The interaction strengths between the processes are shown on the *z*-axis. We can clearly see three different island-like structures in the graph where entities 1 to 5 interact within themselves, 6 to 9 within themselves and 10 to 15 within themselves. The plot clearly shows that there is no cross talk between the entities across different sets even though they are present within the same system.

### Experiments with Real Datasets: Material

We test our method on real biological dataset obtained from in-house microarray experiment designed to measure gene-expression level of around 31,000 genes for Arabidopsis thaliana plant [44]. This section summarizes the experimental details at different stages to obtain the data.

#### Plant growth and leaf material acquisition

Arabidopsis (COL-0) was grown in a controlled environment at 20°C, 70% relative humidity, 250 *μ*mol m-2s-1 light intensity, 16 h day length. Leaf 7 was tagged on emergence and biological replicates were harvested both the morning and evening (7 h and 14 h into light period) at 2 day intervals until fully senescent. This resulted in 22 time point samples from before full expansion to senescent.

#### RNA isolation and probe preparation

RNA was isolated from 4 individual leaves as separate biological replicates using the Triazol method (Invitrogen) followed by RNeasy column purification (Qiagen). RNA was amplified using a MessageAmp II (Ambion) and then labeled with Cy3 or Cy5 using reverse transcriptase (SuperScript II, Invitrogen). Each amplified RNA sample was labeled twice with Cy3 and twice with Cy5 giving 4 technical replicates for each leaf sample. Two Cy3 and C5 labelled samples (in 25% formamide, 5× SSC, 0.1% SDS and 0.5 mg ml-1 yeast tRNA) were mixed in different combinations for hybridization to microarray slides.

#### Microarray analysis

Microarrays (CATMA) carrying 31,000 Arabidopsis gene probes (constructed in house as described in [44]) were hybridized with labeled samples at 42°C overnight. Slides were washed and then scanned using an Affymetrix 428 array scanner at 532 nm (Cy3) and 635 nm (Cy5). Scanned data were quantified using Imagene version 7 software (BioDiscovery, http://www.biodiscovery.com/).

Individual text files quantifying the output for Cy3 and Cy5 were used in the further data analysis.

### Experiments with Real Datasets: Small Example

After testing our method on the synthesized datasets, we test our method on the Arabidopsis data discussed above. We test our method on two samples of different sizes of the same dataset. We first test our method on a smaller sample of 85 genes belonging to three different categories of biological processes. This smaller sample helps us mimic the scenario shown by our synthetic model. The primary advantages of choosing the smaller dataset is that it helps us in minimizing the search space for ontological validation of clusters by mining on-line repositories which may not be complete for all the genes. Later, we apply our technique on a larger dataset of 1800 genes and study the clusters obtained and the general structural properties of the network.

**Figure 5:**
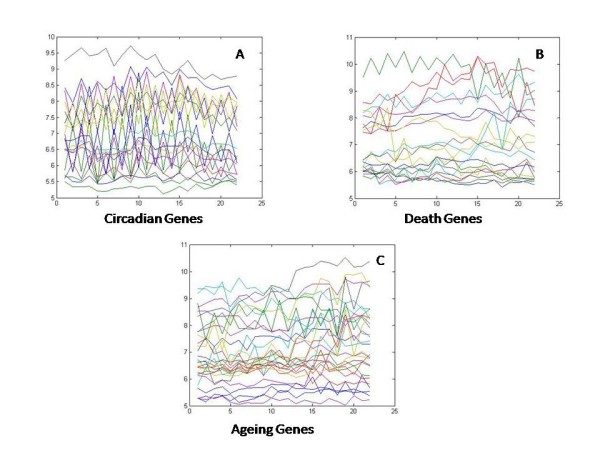
**Temporal profiles of genes selected for smaller dataset for Arabidopsis**. The temporal profiles of the genes selected to constitute the smaller Arabidopsis dataset is shown. A) Genes annotated for circadian activity B) Genes annotated for death and C) Gene annotated for Ageing.

For the smaller dataset, we selected 85 genes belonging to three different categories of biological processes according to the Gene Ontology (GO) database [45]. The selected genes include genes which participate in maintaining the circadian rhythm of the plant, genes which are responsible for aging and the genes involved in plant death. We use the gene ontology (GO) interface provided at the Arabidopsis repository at TAIR http://www.arabidopsis.org/index.jsp to find the names of the genes which are experimentally confirmed to perform above mentioned biological functions. It should be noted that this interface does not provide any *p*-value associated with the GO terms for the selected genes. This selection should be considered just as a weak indication of a gene performing the mentioned biological function. While verifying the results, we use another gene annotation tool (BinGO) [46] which provides the statistical significance for the biological functions for the genes. We selected the time-series data for those genes from our microarray dataset described earlier. Some of the selected genes had at profiles, i.e. the temporal expressions of the genes did not show much fluctuation across time. Such genes were filtered out using the 2*σ *technique and discarded. We finally had a set of 30 genes responsible for circadian rhythm, 34 genes involved in the aging process and 21 genes participating in the cell death, total leading to a set of 85 genes. Figure 5 shows the profiles of the selected genes.

**Figure 6:**
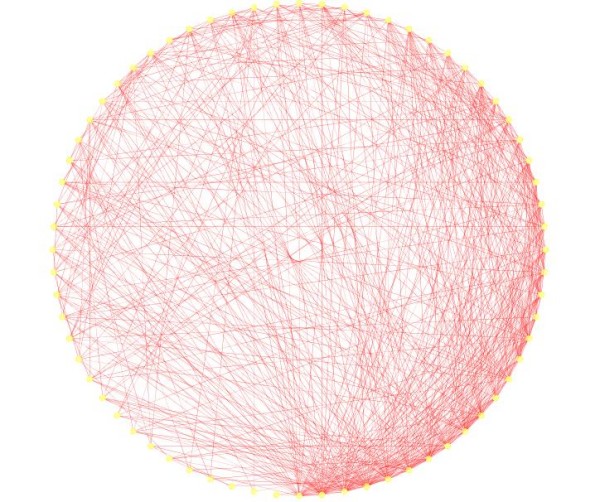
**Degree sorted network structure**. The association graph obtained after applying Granger causality test is displayed in a degree sorted manner.

The temporal profiles of genes were adjusted by taking the first difference of successive time points to obtain the stationary behavior. We then applied the causality test to all the pairs of genes in the system. A complete network with 85 genes has total links equal to 2 × () = 7140. In the second stage, for each 2 pair of node (*X, Y*), we selected the maximum of the causality values for directions *X *→ *Y *and *Y *→ *X *and assigned that value as the weight for the edge between *X *and *Y*. To further simplify the network, we applied a threshold corresponding to 0.975 quantile of all the edge value to select the dominant edges in the network. The final network is presented in Figure 6. The network is arranged in a degree sorted layout.

The vertices with higher degree are bigger in size. The size of a vertex is decided according to the total degree associated with it. The biological relevance of the degree distribution of nodes in a biological network is discussed later in the paper.

**Figure 7:**
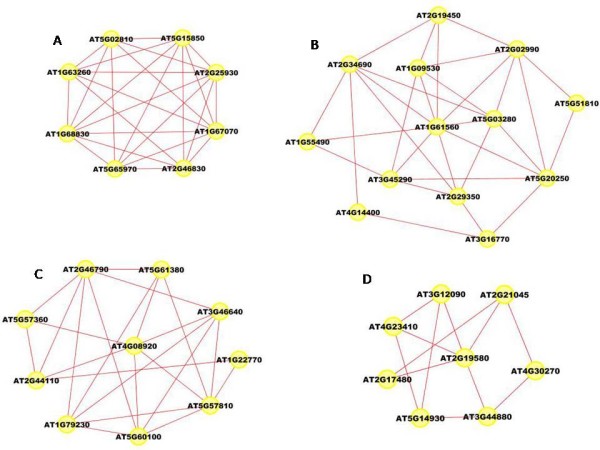
**Extracted subgraphs indicating potential modules of interest in the smaller dataset**. The biological functions performed by modules in respective figures are A.) Circadian rhythm B.) Immune and Defense response C.) Circadian rhythm and D.) Aging. The GO annotations for the genes can be seen in Table 1.

To find the modules in the network, we applied the graph-theoretic approach discussed in the Methods section. The approach detects densely connected regions in the network. Dense regions are the maximally connected sub-components in the graph and may be representative of the complexes in the context of biological networks. The graph-theoretic analysis gives us 4 subgraphs presented in Figure 7. These subgraphs are obtained by setting the *k*-core value = 2 and the results are presented after trimming the nodes with single degree.

To verify our hypothesis that these subgraphs represent functional modules, we use the functional information stored in the Gene Ontology (GO) database using the BinGO tool. Table 1 summarizes the information obtained for all the subgraphs. The first column in the table represents the GO-ID of the functional category stated in the *Functional Description *column. The genes in the table are grouped together to show the GO category they belong to, along with their statistical over-representations in columns 2 and 3. The *p*-values in column 2 are computed by the Hypergeometric Test which is exact and equivalent to an exact Fisher test. To reduce the False Discovery Rates (FDR), a multiple testing correction (Benjamini and Hochberg's FDR correction [47]) is applied and reported in column 3. The Functional Description column lists the biological functions the corresponding genes are associated with. The 'Known/Total' column represents the ratio of genes known to perform a certain biological function in the GO database with respect to the total number of genes having a reference in GO. We can see that the number of known genes in GO are less than the total number of genes submitted. This is due to the fact that the functional annotation of Arabidopsis genomes is incomplete and a particular type of annotation for a gene may differ. We may find a gene that has GO classification and no functional summary text, while other genes have functional summary text and no GO classification, while others have no classification whatsoever.

**Table 1:** Gene ontology details for the networks shown in Figure 7

GO-ID	*p*-value	corr *p*-value	Known/Total	Functional Description	Gene Names
Figure 7(A)					

48511	1.3744E-11	4.1921E-10	4/6	Rhythmic process	AT5G02810, AT2G46830, AT1G68830, AT2G25930
7623	1.3744E-11	4.1921E-10	4/6	Circadian rhythm	AT5G02810, AT2G46830, AT1G68830, AT2G25930

Figure 7(B)					

9814	4.5406E-11	7.6281E-9	5/8	Defense response	AT1G55490, AT2G34690, AT5G03280, AT1G61560, AT4G14400
45087	2.5439E-10	2.1369E-8	5/8	Innate immune response	AT1G55490, AT2G34690, AT5G03280, AT1G61560, AT4G14400
6955	3.8828E-10	2.1743E-8	5/8	Immune response	AT1G55490, AT2G34690, AT5G03280, AT1G61560, AT4G14400
2376	5.7329E-10	2.4078E-8	5/8	Immune system process	AT1G55490, AT2G34690, AT5G03280, AT1G61560, AT4G14400
8219	3.9627E-9	1.1096E-7	4/8	Cell death	AT1G55490, AT2G34690, AT5G03280, AT4G14400
16265	3.9627E-9	1.1096E-7	4/8	Death	AT1G55490, AT2G34690, AT5G03280, AT4G14400

Figure 7(C)					

7623	1.6563E-14	9.8551E-13	5/7	Circadian rhythm	AT5G57360, AT2G46790, AT1G22770, AT5G61380, AT4G08920
48511	1.6563E-14	9.8551E-13	5/7	Rhythmic process	AT5G57360, AT2G46790, AT1G22770, AT5G61380, AT4G08920

Figure 7(D)					

16280	3.0760E-13	1.4149E-11	5/6	Aging	AT3G12090, AT4G23410, AT5G14930, AT2G19580, AT2G21045
32502	1.1218E-8	2.5802E-7	6/6	Developmental process	AT3G12090, AT4G23410, AT3G44880, AT5G14930, AT2G19580, AT2G21045

The subgraph in Figure 7(a) is composed of 8 genes (AT5G02810, AT1G68830, AT1G63260, AT2G46830, AT5G65970, AT5G15850, AT1G67070, AT2G25930). 6 out of the 8 genes are known in the GO database. No annotations could be obtained for the remaining 2 genes (AT5G65970 and AT1G67070). 4 out of the 6 known genes are clearly known as the genes participating in the circadian rhythm process. AT5G15850 is known to be associated with the regulation of flower development which is related to the circadian rhythm of the Arabidopsis plant. Gene AT1G63260 is wrongly classified as it is known to participate in the aging process. Similarly, in the second network (Figure 7(b)), there are 13 genes in all(AT1G09530, AT4G14400, AT2G19450, AT2G02990, AT5G51810, AT5G20250, AT3G16770, AT2G29350, AT3G45290, AT1G55490, AT1G61560, AT2G34690, AT5G03280). 8 of the genes have entries in GO and no annotation could be found for the remaining 5 genes (AT1G09530, AT5G51810, AT5G20250, AT3G45290 and AT3G16770). 5 out of the 8 known genes are involved with the biological process of defense, immune response and cell death. AT2G19450 and AT2G02990 are known for 'response to stress'(GO process ID - 9651). Gene AT2G29350 is classified for 'aging' and is the odd member in the network. The third network shown in Figure 7(c) has 10 genes (AT2G44110, AT5G61380, AT4G08920, AT5G57360, AT2G46790, AT5G60100, AT1G79230, AT3G46640, AT5G57810, AT1G22770) with 7 of them known in the GO database and 3 (AT3G46640, AT1G79230, AT2G44110) are without any annotation. 5 out of the 7 annotated genes are known to participate in rhythmic activity. Gene AT5G60100 is known for regulation of circadian rhythm (GO process ID - 42752). Gene AT5G57810 is known for 'aging' and is wrongly put in this network. The last subnetwork shown in Figure 7(d) is composed of 8 genes (AT4G23410, AT5G14930, AT3G44880, AT4G30270, AT2G17480, AT2G21045, AT2G19580, AT3G12090). 6 out of the 8 genes are known in the GO database. All the 6 genes are known to participate in aging process of the plant. No annotations were found for genes AT2G17480 and AT4G30270.

### Experiments with Real Datasets: Bigger Example

**Figure 8:**
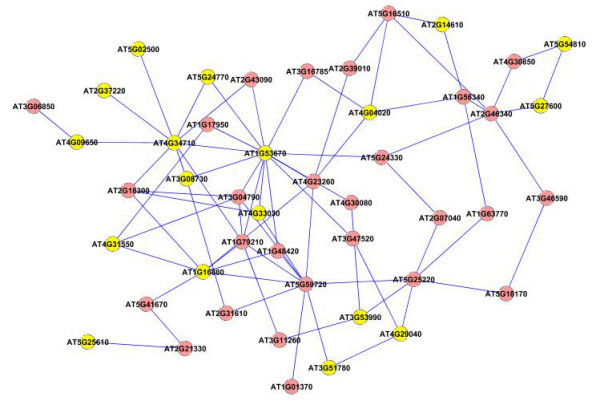
**Extracted subgraph indicating potential module of interest in the bigger dataset - Set 1**. The genes belonging to *Response to stress *category are highlighted in yellow. The GO annotations of the highlighted genes are presented in Table 2.

**Figure 9:**
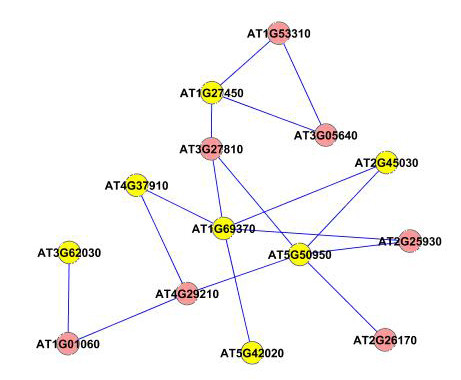
**Extracted subgraph indicating potential module of interest in the bigger dataset - Set 2**. The genes belonging to *Cytoplasmic part *category are highlighted in yellow. The GO annotations of the highlighted genes are presented in Table 2.

**Figure 10:**
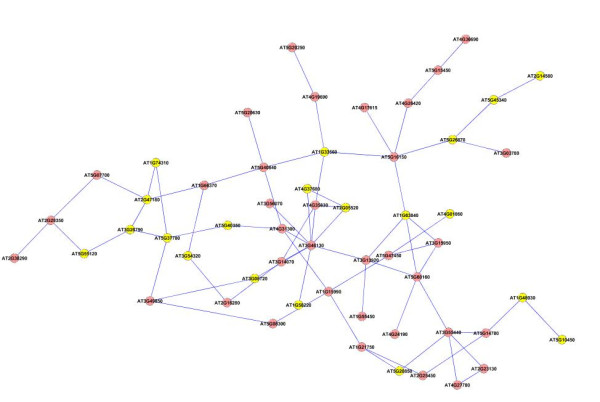
**Extracted subgraph indicating potential module of interest in the bigger dataset - Set 3**. The genes belonging to *Response to stimulus *category are highlighted in yellow. The GO annotations of the highlighted genes are presented in Table 2.

**Figure 11:**
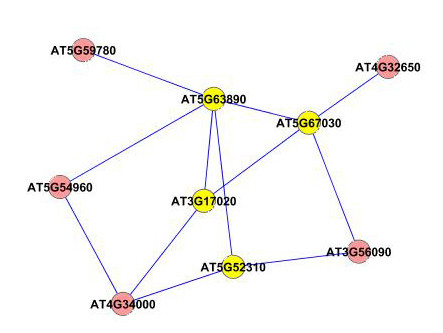
**Extracted subgraph indicating potential module of interest in the bigger dataset - Set 4**. The genes belonging to *Response to abiotic stimulus *category are highlighted in yellow. The GO annotations of the highlighted genes are presented in Table 2.

**Figure 12:**
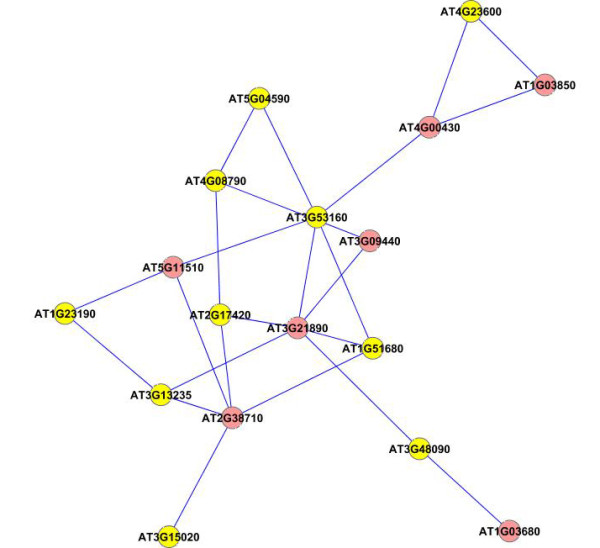
**Extracted subgraph indicating potential module of interest in the bigger dataset - Set 5**. The genes belonging to *Catalytic activity *category are highlighted in yellow. The GO annotations of the highlighted genes are presented in Table 2.

**Figure 13:**
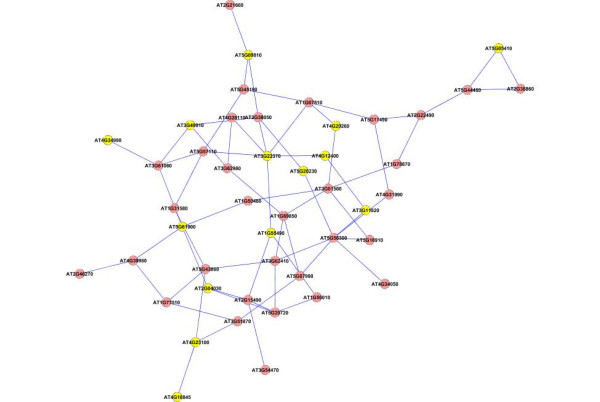
**Extracted subgraph indicating potential module of interest in the bigger dataset - Set 6**. The genes belonging to *Response to stress *category are highlighted in yellow. The GO annotations of the highlighted genes are presented in Table 2.

**Figure 14:**
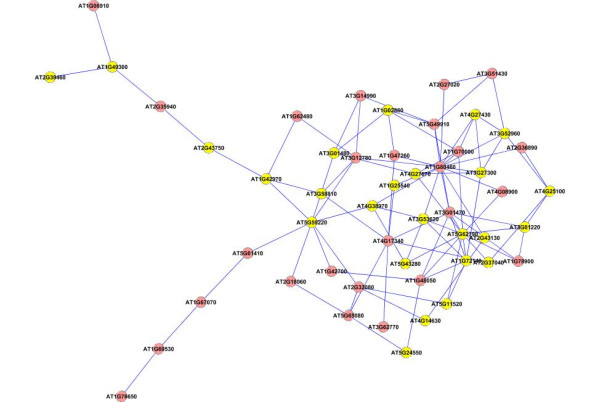
**Extracted subgraph indicating potential module of interest in the bigger dataset - Set 7**. The genes belonging to *Cell part *category are highlighted in yellow. The GO annotations of the highlighted genes are presented in Table 2.

We next applied our method on a larger dataset of 1800 genes selected according to their frequency profiles (also discussed in [48]). We ranked the genes according to their power spectrum in frequency domain by taking a Fast Fourier Transformation of the data, and chose the top 1800 genes for analysis with our method. We constructed an association network for all the pairs of genes using the test for causality to detect the edges in the network. We applied a threshold corresponding to 0.99 percentile of all the edge values to select the most dominant edges in the network for further analysis. We applied the dense region finding method on the network using different combinations of *k*-core score which resulted in a number of different clusters. We present some of the clusters we found in Figures 8, 9, 10, 11, 12, 13 and 14. The GO descriptions of selected genes in the shown clusters is summarized in Table 2. The table reports the information in the same manner as it did in case of the smaller sized data sample.

**Table 2:** GO annotations for the highlighted genes shown in Figures 8-14

GO-ID	*p*-value	corr *p*-value	Known/Total	Functional Description	Gene Names
Figure 8					

6950	5.1715E-13	5.6542E-11	18/38	Response to stress	AT3G08730, AT5G27600, AT4G33030, AT1G53670, AT2G37220, AT4G34710, AT4G31550, AT5G54810, AT4G09650, AT4G29040, AT5G24770, AT2G14610, AT3G51780, AT3G53990, AT4G04020, AT1G16880, AT5G25610, AT5G02500

Figure 9					

44444	3.5066E-4	2.0147E-2	7/11	Cytoplasmic part	AT5G42020, AT3G62030, AT1G27450, AT4G37910, AT2G45030, AT5G50950, AT1G69370

Figure 10					

51869	5.5701E-12	2.3450E-9	20/41	Response to stimulus	AT5G20850, AT5G55120, AT3G08720, AT4G37680, AT5G26870, AT1G33560, AT2G47180, AT2G05520, AT1G48030, AT4G01060, AT5G37780, AT1G63840, AT2G14580, AT1G58220, AT3G26790, AT3G54320, AT5G10450, AT1G74310, AT5G45340, AT5G40350

Figure 11					

9628	1.1048E-5	1.3147E-3	4/7	Response to abiotic stimulus	AT5G52310, AT3G17020, AT5G67030, AT5G63890

Figure 12					

3824	9.0400E-4	4.2857E-2	10/15	Catalytic activity	AT2G17420, AT3G15020, AT5G04590, AT3G13235, AT1G23190, AT3G53160, AT3G48090, AT4G23600, AT4G08790, AT1G51680

Figure 13					

6950	7.0271E-10	6.6055E-8	14/37	Response to stress	AT5G20230, AT5G61900, AT4G16845, AT3G22370, AT2G04030, AT1G55490, AT3G11820, AT4G12400, AT4G34990, AT4G23100, AT4G20260, AT3G49910, AT5G09810, AT5G05410

Figure 14					

44464	2.6470E-3	1.8771E-2	25/36	Cell part	AT4G27670, AT5G59220, AT4G25100, AT3G58810, AT4G14630, AT3G53620, AT5G11520, AT3G27300, AT1G42970, AT5G43280, AT4G27430, AT1G49300, AT2G39460, AT2G37040, AT3G01480, AT5G24550, AT1G72140, AT5G62790, AT1G25540, AT1G02860, AT4G38970, AT2G43130, AT3G52960, AT3G01220, AT2G43750

#### Simple network statistics

We computed certain network statistics to confirm that our network is not a randomly generated network and has the properties desired in a biological network. A total of 1353 nodes were present in the network after filtering out weaker edges. The total number of edges present in the network was 21,214 which is around 1.1% of the total possible directed edges in the network, which is an indication of sparseness, a common characteristics of biological networks [49]. There is one connected component in the network indicating strong connectivity. The mean shortest path length is 2.6 which means that most genes are close to each other and the network diameter representing the maximum distance between two connected nodes is 6. Both the phenomenon have been described as small world properties of real networks [50]. We also compute and report the following widely used topological properties for our network.

#### Node degree distribution

**Figure 15:**
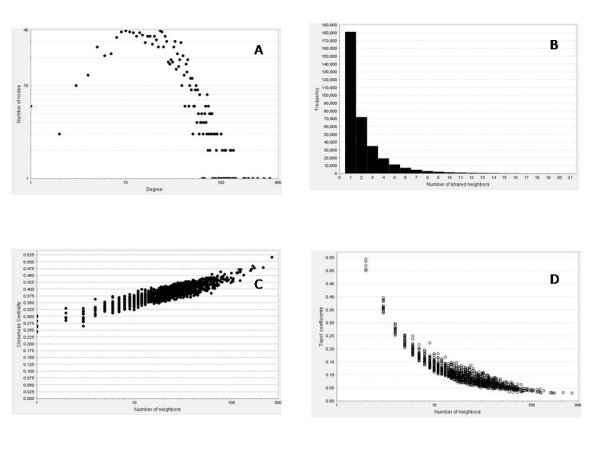
**Structural properties of association network obtained for bigger dataset**. A) A power-law like distribution obtained for the node degree distribution. B) A distribution of number of partners shared between a pair of nodes C) Closeness centrality of all the nodes D) Plot for topological coefficient.

We calculated the degree distribution *p*(*k*) of the genes, measuring the probability that a given gene interacts with *k *other genes. Barabasi and Albert [49] used the node degree distribution to distinguish between the topologies of random and scale-free networks. Our network shows a power-law like distribution on log scale as shown in Figure 15(a). The plot shows that there are few nodes with large number of neighbors and they dominate the connectivity in the network. Also, the tail of power-law distribution on normal scale indicates that highly connected vertices have a large degree of occurring. Such networks exhibit preferential connectivity indicating that a new node will link to established nodes which are well connected, resulting in a structure where few hubs hold together numerous small nodes.

#### Shared neighbor distribution

Figure 15(b) shows the shared neighbor distribution for the network. *P*(*i*, *j*) is the number of partners shared between nodes *i *and *j*, that is, nodes that are neighbors of both *i *and *j*. The shared neighbors distribution gives the number of node pairs (*i*, *j*) with *P*(*i*, *j*) = *k *for *k *= 1,2, 3.... The distribution again shows a power law like distribution indicating the presence of motifs with large numbers of connected components in the network.

#### Closeness centrality

Closeness centrality is a measure of how fast information flows from a given node to other reachable nodes in the network. Closeness centrality (*C*) of a network with *n *nodes is computed as the reciprocal of the average shortest path length is computed as follows: *C*(*n*) =  where *L*(*i*, *j*) is the length of the shortest path between two nodes *i *and *j*. Figure 15(c) plots the closeness centrality of all the nodes against number of neighbors. The isolated nodes have their closeness centrality equal to 0. An increasing trend of closeness centrality in our network further indicates strong connectivity and ability to form hubs.

#### Topological coefficient

Another characteristics of interaction networks can be captured by calculating the topological coefficients [51,52]. The topological coefficient, *TC*(*k*), is a relative measure for the extent to which a gene in the network shares interaction partners with other genes. Also the topological coefficient as shown in Figure 15(d) decreases with the number of links (close to ), demonstrating that, relatively, in our network, hubs do not have more common neighbors than genes with fewer links. This indicates that genes with many links are not artificially clustered together. Moreover, it confirms the presence of modular structures in the network organization.

### Comparison With Respect to Other Existing Methods

In order to have a comparison of our proposed method with some existing methods, we use the synthetic datasets and the smaller Arabidopsis dataset of 85 genes discussed in the earlier sections. We apply two widely used techniques to establish association between the pairs of genes in the dataset. The association between genes are measured using a) the Pearson correlation coefficient and b) the Euclidean distance. First, we computed the correlation coefficients and the Euclidean distances for the node pairs in the synthetic datasets. The results are presented in Table 3 and 4 respectively. We can see in Table 3 that most of the correlation coefficients for the datasets are very low in magnitude. For Dataset 1, only the links between Node 2 and Node 3, Node 2 and Node 4, and Node 3 and Node 4 have highest magnitude close to 0.75, the rest of the links show very weak correlation. Similarly, in Dataset 2, only the links between Node 2 and Node 4 exhibit higher correlation of 0.6 compared to the correlation shown by other node pairs in the dataset. In Dataset 3, we have no pair of node showing any significant correlation. The analysis of synthetic datasets using Euclidean distance is presented in Table 4. It can be seen from the values in Table 4 that the Euclidean distance measure fails to give any clear indication of association between nodes in any dataset. Our method of Granger causality test produced much superior results compared to these methods as we saw in the *Results and Discussion *section. We then extend our comparison to the smaller Arabidopsis dataset of 85 genes.

**Table 3:** The correlation matrix for synthetic datasets 1, 2 and 3.

Dataset 1						
**Node**	**1**	**2**	**3**	**4**	**5**	

1	1.0000	0.2613	-0.2309	-0.2500	0.0871	
2	0.2613	1.0000	-0.7114	-0.7515	0.1351	
3	-0.2309	-0.7114	1.0000	0.7654	-0.1283	
4	-0.2500	-0.7515	0.7654	1.0000	-0.3125	
5	0.0871	0.1351	-0.1283	-0.3125	1.0000	

Dataset 2						

Node	1	2	3	4		

1	1.0000	-0.0944	0.0621	-0.1088		
2	-0.0944	1.0000	-0.0940	0.6040		
3	0.0621	-0.0940	1.0000	0.0024		
4	-0.1088	0.6040	0.0024	1.0000		

Dataset 3						

Node	1	2	3	4	5	6

1	1.0000	0.1872	0.0449	0.0329	0.1118	0.1531
2	0.1872	1.0000	0.1105	0.0292	0.0748	0.3101
3	0.0449	0.1105	1.0000	-0.0001	0.2516	0.0665
4	0.0329	0.0292	-0.0001	1.0000	0.0821	0.2282
5	0.1118	0.0748	0.2516	0.0821	1.0000	0.0907
6	0.1531	0.3101	0.0665	0.2282	0.0907	1.0000

**Table 4:** The Euclidean distance matrix for synthetic datasets 1, 2 and 3.

Dataset 1						
**Node**	**1**	**2**	**3**	**4**	**5**	

1	0	50.6180	60.5454	66.3305	53.7858	
2	50.6180	0	49.0080	57.3540	35.0406	
3	60.5454	49.0080	0	21.1572	36.9004	
4	66.3305	57.3540	21.1572	0	46.7355	
5	53.7858	35.0406	36.9004	46.7355	0	

Dataset 2						

Node	1	2	3	4		

1	0	57.2707	49.4072	54.0319		
2	57.2707	0	35.4161	23.3695		
3	49.4072	35.4161	0	28.6682		
4	54.0319	23.3695	28.6682	0		

Dataset 3						

Node	1	2	3	4	5	6

1	0	32.1456	32.0493	31.7813	33.2172	34.6842
2	32.1456	0	25.0916	25.6146	28.3732	27.0407
3	32.0493	25.0916	0	21.9953	22.6557	28.4800
4	31.7813	25.6146	21.9953	0	24.4613	25.7756
5	33.2172	28.3732	22.6557	24.4613	0	30.6190
6	34.6842	27.0407	28.4800	25.7756	30.6190	0

**Figure 16:**
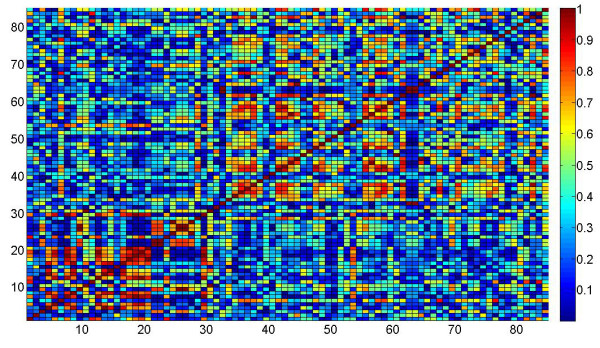
**Correlation matrix for smaller Arabidopsis dataset**. The association matrix obtained using Pearson correlation for the smaller Arabidopsis dataset is shown. The strengths of interactions between genes are quantified according to the color-map presented in the figure.

**Figure 17:**
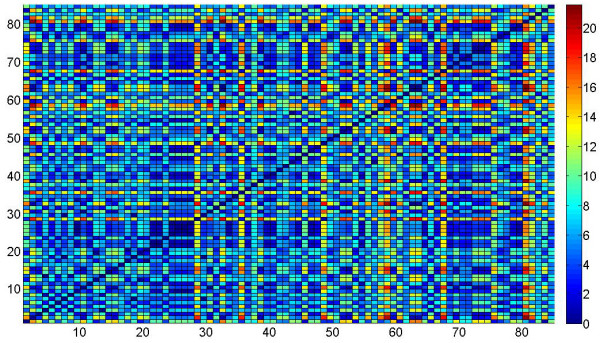
**Distance matrix for smaller Arabidopsis dataset**. The association matrix obtained using Euclidean distance for the smaller Arabidopsis dataset is shown. The strengths of interactions between genes are quantified according to the color-map presented in the figure.

The small size and the knowledge about the functionality of genes are the main advantages of using the smaller dataset for Arabidopsis. The small size of dataset also allows us to present the results in an easy-to-view graphical format. The genes in the dataset were arranged in an ordered fashion before computing the association between them, i.e., the first 30 in the dataset of 85 genes preformed circadian rhythm related activity, the next 34 genes were associated with aging, and the last 21 genes participated in cell death. Figure 16 and Figure 17 present the graphical representation of the association matrices obtained for the gene pairs using correlation coefficient and Euclidean distance respectively. Each cell in an association matrix is filled with a color based on the quantitative entry in that cell. The mapping of colors with the magnitudes of cells is displayed by the color-bars in the figures. We can see that the color coding starts from blue (for low magnitude of association) to red (for high magnitude of association). The strongly associated gene pairs are represented by shades of red in their respective cells. The diagonal entries of both the association matrices are drawn in dark red, indicating maximum degree of association between self-to-self pair. The association matrices are symmetric, thus, the inspection of only the lower diagonal entries should suffice in detection of strongly associated gene pairs.

In an ideal scenario, where the genes performing similar activity group together, we expect three distinct regions in Figures 16 and 17. The lower diagonal blocks from cell 1 to 30, cell 31 to 64, and cell 65 to 85 should indicate a high degree of intra-block association, each block should be colored in different shades of red according to the color-magnitude mapping shown in the color-bars. But, this is not the case in the figures obtained by us where we can see no clear blocks in the figures. The lack of any block-wise patterns in the color coded cells of association matrices indicate the absence of strong associative information between genes based on the measures discussed above. This is the first indication that the measures like correlation and euclidean distance may not be suitable for our dataset.

**Figure 18:**
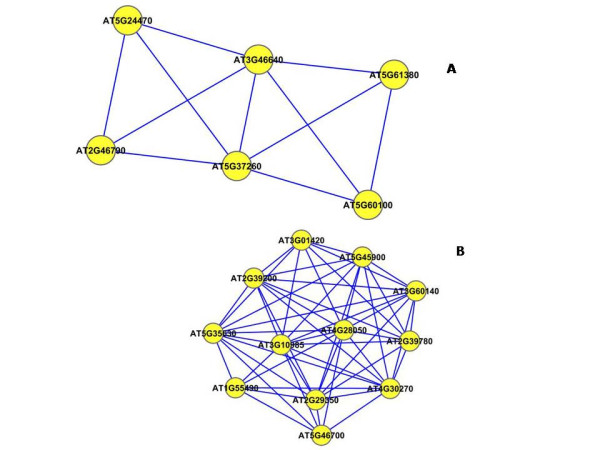
**Subgraphs obtained by using correlation as a measure of association in the smaller Arabidopsis dataset**. Two subgraphs of potential interest were detected when correlation coefficient was used to establish association between genes in the smaller Arabidopsis dataset. The GO annotation of recognised genes are presented in Table 5.

To investigate further, we applied a threshold to keep the strongest edges in the graphs obtained from the association matrices. The criteria to choose the threshold for selecting the strong edges in the graphs was same as the one used before in case of smaller Arabidopsis dataset. The filtered graphs were analyzed using the graph-theoretic technique with the similar settings as used before. The correlation based associative graph resulted in two subgraphs shown in Figure 18, whereas the euclidean distance based graph did not yield any subgraph at all. The gene ontology analysis of the two subgraphs shown in Figure 18 is presented in Table 5. We can see that in Network 1, three out of total six genes belonged to rhythmic process related activity, whereas, in Network 2, five out of total of twelve genes belonged to aging process. These networks and their related biological relevances are much inferior compared to the subgraphs obtained in section using our technique, where we obtained 4 distinct subgraphs with distinct biological functions and better gene ontology results.

**Table 5:** GO annotations for clusters found in the smaller Arabidopsis dataset using correlation as the measure of association between genes

GO-ID	*p*-value	corr *p*-value	Known/Total	Functional Description	Gene Names
Figure 18(A)					

48511	4.0370E-9	8.2759E-8	3/4	Rhythmic process	AT5G24470, AT2G46790, AT5G61380

Figure 18(B)					

16280	2.8611E-12	2.0600E-10	5/8	Aging	AT5G45900, AT2G29350, AT5G35630, AT3G10985, AT4G28050

We have used a fresh and distinct approach to cluster temporal microarray gene expression data. One of the key questions that we have tried to address using this method is that how some variables are useful for *forecasting *others. The proposed method facilitates a way to study such forecasting relationships between two variables. In other words, we are asking if a variable *X *can predict another variable *Y*. Equivalently, we can say if *X *is exogenous in time-series sense with respect to *Y *or not. Yet a third expression meaning the same thing is, if *X *is linearly informative about *future Y*. The basic idea behind this method is, if an event *X *causes another event *Y*, then *X *should *precede Y *in time. This is why our illustrative models are based on time, and within that time frame the lags like *t *- 1, *t *- 2, ⋯ etc. denote the temporal association within the processes.

While discussing widely used pairwise association methods for clustering, like any form of correlation or distance based methods, the time is static. In these methods, the time does not play any role. The core of these methods rely on association rather than prediction. So if we re-order the sequence of observations for any pair of variables (*X*, *Y*), the association measure between them does not change. As for example, let the original observation be *X *= {*x*_*t*-1_, *x*_*t*-2_, *x*_*t*-3_} and *Y *= {*y*_*t*-1_, *y*_*t*-2_, *y*_*t*-3_}. The Association measure using correlation/distance for (*X, Y*) = *C*. After reordering of the observations, let *X' *= {*x*_*t*-3_, *x*_*t*-1_, *x*_*t*-2_} and *Y' *= {*y*_*t*-3_, *y*_*t*-1_, *y*_*t*-2_}. The new association measure using correlation/distance for (*X', Y'*) = *C' *where *C *= *C'*. Hence, this assumption is not suitable for dynamical systems. This is the reason why the usual pairwise association methods can give us less reliable results than the ones by our method. And hence a comparison between the two methods will not be fair. There has been some work in model based clustering methods based on Bayesian statistics where the dynamics of profiles (modeled as regressive processes) have been used to create clusters [39,53]. Such methods are different from our approach as, first, our approach is based on the frequentist approach rather than the Bayesian approach, and second, the essence of our approach lies in detecting the causal association between genes. Another important aspect to consider is the choice of time lag in our method which is decided using the AIC criteria. The lag value is not fixed, but is chosen iteratively for each individual pair (*X*, *Y*) according to what describes the variables best.

We have demonstrated the performance of the method using various artificial datasets and examples from real biological datasets. It is easy to see that the pair-wise association based techniques, like distance or correlation based measures, would not work as desired, when we are investigating a system where the interaction with respect to time is an important concept.

## Conclusions

Clustering helps in reducing the data dimensions by grouping genes with similar profiles or similar functionalities. In this paper, we proposed a clustering method to group functionally related genes in a temporal microarray dataset. Our method exploits the temporal interdependence between genes. The interdependence was determined using the test of Granger causality between two time series. The method is simple in its implementation, and testable at every stage. We analyze the association graph using a graph-theoretic method to detect the dense regions in the graph. These dense regions could be indicators of potential biological complexes and motifs. The graph-theoretic approach helps us in detecting the functionally interesting regions in a large network derived by the Granger causality test. We test our approach using a set of artificial datasets and two datasets of different sizes belonging to the Arabidopsis experiment. The functional similarity between genes belonging to detected clusters was verified using the publicly available gene ontology database. We further analyzed the structural properties of the association network obtained for the larger of the two datasets for Arabidopsis. We show using different network characteristics that the computed association network is not a random network in its structure, and has the properties expected in a real biological network.

There are few considerations which should be taken into account while applying this approach. The data must be cleaned properly using appropriate normalization method to remove unwanted experimental biases. For any time-series based statistical method, it is important that the data has been collected at intervals which capture the natural changes in the system. Selection of correct lag order using an information based criteria is also important as the test of Granger causality is strongly dependent on that decision. Due to the small number of time points in our dataset, we have used linear form of Granger causality to establish relationship between genes in our model. It should be noted that Granger causality test is not restricted to only linear models, and it can be readily extended to include non-linear terms in case we observe any non-linear behavior in the data. Some examples of non-linear extension if Granger causality can be found in the publication by Ancona et al. [54] and Marinazzo et al. [55]. Most important of all, the experimental design should be able to support the hypothesis of the practitioner. Further care should be taken while discovering *directional *causal links using Granger causality [9,10]. It should be noted that multivariate approaches instead of pair-wise to detect interactions between genes can give better result while *re-engineering a causal network structure *from data [11,48,56,57]. In this paper, our effort was not to detect a *causal *network structure from gene data, but to find a suitable association matrix based on interactions between them. Once the interesting modules have been found, different reverse engineering methods like Bayesian networks, Structural equations etc. can be applied to infer causal networks from selected genes of interest.

## Methods

### Inference of Causal Association

In accordance to general equilibrium theory, economists assume that everything depends on everything else; and hence, the notion of causal relationship between different time-series arises. The idea of causality is related to the idea of succession in time and that the cause always precedes the effect. Consider two processes *X *and *Y*. If *Y *is causal to *X*, the current and lagged values of *Y *should contain information that can be used to improve the forecast of *X*, rather than considering only the past and present values of *X *alone. Granger [8] proposed the definition of causality, widely known as Granger-causality in the literature to examine whether the forecast of future values of *X *can be improved if along with *X*'s own values - the current and past values of *Y *are also taken into account. Another reason why lagged values are considered for corresponding variables is to avoid spurious regressions between dependent and explanatory variables [58]. The inclusion of past values of both variables implies that the time-series are filtered. With respect to the causal relationship between two time-series, only the corresponding innovations matter [59]. We assume that our time-series is stationary in nature. Let *I*_*t *_be the total information present at time *t*. *I*_*t *_contains two time series *X *and *Y*. Let  be the set of all current and past values of *X*_*t *_i.e.  = {*x*_*t*_, *x*_*t*-1_, ...} and similarly  = {*y*_*t*_, *y*_*t*-1_, ...}. Let *σ*^2^(.) be the variance of the corresponding forecast error. Granger's definition of causality between *X *and *Y *included three scenarios.

1. Granger Causality: *Y *is Granger causal to *X *if and only if the future values of *X *can be predicted better i.e with a lower variance, if the current and past values of *Y *are used.

2. Instantaneous Granger Causality: *Y *is instantaneously Granger causal to *X *if and only if the application of an optimal linear function leads to the better prediction of future value of *X*, *x*_*t*+1 _if the future value of *Y*, *y*_*t*+1 _is used in addition to the current and past values of *Y*.

3. Feedback: The feedback between *X *and *Y *exists if *X *is causal to *Y *and *Y *is causal to *X*.

Feedback is only defined for the case of simple causal relations because the direction of instantaneous causality cannot be determined without additional information or assumption.

The bidirectional Granger causality can be tested in the context of linear regressive models. For a pairwise interaction between two variables, we use autoregressive specification of a bivariate vector autoregression. Assume a particular autoregressive lag length *p*, and we can estimate the following unrestricted equation by ordinary least squares (OLS):(1)

where *X*_*t *_is the is the prediction of the *X *at time *t *based on its own past values as well as the past values of *Y*, *α*_*i *_and *β*_*i *_are the weighting factors, and *u*_*t *_is the prediction error(residual) with a variance that measures the strength of the prediction error. If all the weighting factors *β*_*i *_in Equation (1) are equal to zero then we can conclude that *Y *does not contribute towards the prediction of *X*, but in the case of any *β*_*i *_being not equal to zero, we will say that the past values of *Y *are contributing towards the prediction of the current *X*. Therefore we can have two hypotheses as follows -(2)

We can conduct a F-test of the hypotheses by estimating the following equation using Ordinary Least Squares(4)

where ϵ_*t *_is the prediction error or residual.

Let *RSS*_1 _and *RSS*_0 _be the sum of squared residuals of Equation (1) and (4), respectively, i.e.(5)

and(7)

If the test statistic *S *is greater than the specified critical value specified critical value, we reject the null hypothesis that *Y *does not Granger-cause *X*.

The results are strongly dependent on the number of lags of explanatory variables. To find a suitable lag value in Equations (1) and (4) we use Akaike Information Criteria(AIC, [60]). Any value *p *which minimizes the AIC value is chosen as the lag order.(8)

where *σ *is the estimated noise covariance, *m *is the dimension of the stochastic process and *n *is the length of the data window used to estimate the model.

We will use the test of Granger causality to establish association between gene pairs in our interaction network. If the test for causality passes in any direction, either from *X *→ *Y *or from *Y *→ *X*, we add an edge in the network. We are not interested in the direction of the edge and the association network is not directional at all.

### Detection of Dense Regions in Association Graph

Even though most of the biological networks are sparse in their connectivity, the complexity of connections increases with the increasing number of nodes. A network of interacting entities can be readily modeled as a graph where the entities are represented by nodes and the associations between them as edges. It is often argued [61,62] that graph theoretic approaches can help analyze large interacting networks to find clusters (highly dense regions) in a network. Clusters in a gene-gene interaction network are often biological complexes or part of biochemical pathways [63]. Algorithms for finding clusters or highly dense regions are an ongoing topic of research and are often based on network flow theory [64] or spectral clustering [29]. We use a clustering method proposed by Bader and Hogue [65] to detect the dense regions in the association network obtained by our Granger causality based method. The method weighs all the vertices based on their local network density to detect dense regions in the graph. The decision to use this algorithm to analyze our association matrix was based on two reasons: a) this is one of the earliest methods to use a clustering algorithm to identify molecular complexes in a biological network, and hence is widely known, and, b) it has a publicly available software plug-in for a widely used network analysis platform called Cytoscape [61]. Thus, the method and its implementation are both widely used and tested. It should be noted that application of other clustering methods to detect dense regions can produce different clusters and some may have better performances but these are not tested here.

The functioning of the method by Bader and Hogue can be understood in the following way. Given a graph *G *= (*V*, *E*), where *V *and *E *being the sets of vertices and edges respectively, the density of a graph is based on the connectivity level and is defined as *D*_*G *_= |*E*|/|*E*_*max*_|, where *E*_*max *_is the total number of all possible edges in a complete graph *G*.

The vertex weighting in the graph starts by weighing all the vertices based on their local network density using the highest *k*-core of the vertex neighborhood. A *k*-core is a graph of minimal degree, ∀*v *∈ *V *and the degree of *v *≥ *k*. The highest *k*-core of a graph is the central and most densely connected subgraph. The highest *k*-core component gives us the highest *k*-core level, *k*_*max *_in the vertex neighborhood. The final weight of the vertex is the product of *k*_*max *_and the density of the corresponding highest *k*-core component.

This type of weighting amplifies the weighting of heavily connected graph regions while removing the less connected graph regions which are present in abundance.

Once the vertex weighting is done, the algorithm seeds a subgraph(complex) with highest weighted vertex and moves outwards to include vertices in the neighborhood whose weight is greater than a given threshold. The algorithm propagates through the included neighbors and recursively checks the subsequent nodes. The process stops when no more nodes can be added to the complex and is repeated for the next highest unseen weighted vertex in the network.

In the post-processing stage, the complexes which do not contain at least 2-core (graph with minimum degree 2) are filtered out. Finally, all the complexes in the network are scored and ranked. The complex score for a given subgraph *G*_*C *_= (*V*_*c*_, *E*_*c*_) is defined as the product of the density of the subgraph and the number of vertices (*D*_*c *_× |*V*_*c*_|). Other scoring schemes are also possible but are not tested in the original algorithm.

## Authors' contributions

RK and CL conceived and designed the study. VBW performed the experiments. RK analyzed the data. All authors have read and approved the final manuscript.

## References

[B1] Kim BR Littell RC Wu RL Clustering the periodic pattern of gene expression using Fourier series approximations Curr Genomics 2006 7 197 203 10.2174/138920206777780229

[B2] Harmer SL Hogenesch JB Straume M Chang HS HB Orchestrated transcription of key pathways in Arabidopsis by the circadian clock Science 2000 290 2110 2113 10.1126/science.290.5499.2110 11118138

[B3] Wichert S Fokianos K Strimmer K Identifying Periodically Expressed Transcripts in Microarray Time Series Data Bioinformatics 2004 20 5 20 10.1093/bioinformatics/btg364 14693803

[B4] Quackenbush J Computational analysis of microarray data Nat Rev Genet 2001 2 6 418 427 10.1038/35076576 11389458

[B5] Speed T Statistical Analysis of Gene Expression Microarray Data 2003 Chapman and Hall/CRC

[B6] Kerr MK Churchill GA Statistical design and the analysis of gene expression microarray data Genet Res 2001 77 123 128 11355567 10.1017/s0016672301005055

[B7] Androulakis IP Yang E Almon RR Analysis of Time-Series Gene Expression Data: Methods, Challenges and Opportunities Annual Review of Biomedical Engineering 2007 9 205 228 10.1146/annurev.bioeng.9.060906.151904 17341157 PMC4181347

[B8] Granger C Investigating causal relations by econometric models and cross-spectral methods Econometrica 1969 37 424 438 10.2307/1912791

[B9] Mukhopadhyay N Chatterjee S Causality and pathway search in microarray time series experiment Bioinformatics 2007 23 442 449 10.1093/bioinformatics/btl598 17158516

[B10] Nagarajan R Upreti M Comment on causality and pathway search in microarray time series experiment Bioinformatics 2008 24 7 1029 1032 10.1093/bioinformatics/btm586 18304931 PMC2679500

[B11] Krishna R Guo S A partial granger causality approach to explore causal networks derived from multi-parameter data Lecture notes in Computer Science 2008 5307 9 27 full_text

[B12] Guo S Wu JH Ding MZ Feng JF Uncovering interactions in the frequency domain PLoS Comp Biology 2008 4 5 e1000087 10.1371/journal.pcbi.1000087 PMC2398781 18516243

[B13] Jeong H Mason SP Barabsi AL Oltvai ZN Lethality and centrality in protein networks Nature 2001 411 41 42 10.1038/35075138 11333967

[B14] Tanay A Sharan R Kupiec M Shamir R Revealing modularity and organization in the yeast molecular network by integrated analysis of highly heterogeneous genomewide data PNAS 2004 101 2981 2986 10.1073/pnas.0308661100 14973197 PMC365731

[B15] Barabsi A Linked: The New Science of Networks 2002 Basic Books

[B16] DHaeseleer P How does gene expression clustering work? Nat Biotechnol 2005 23 12 1499 1501 10.1038/nbt1205-1499 16333293

[B17] Seber GAF Multivariate Observations 1984 John Wiley & Sons Inc

[B18] Eichler G Huang S Ingber D Gene expression dynamics inspector (GEDI): for integrative analysis of expression profiles Bioinformatics 2003 19 17 2321 22 10.1093/bioinformatics/btg307 14630665

[B19] Johnson R Wichern D Applied multivariate statistical analysis 1988 Prentice-Hall

[B20] Eisen M Spellman P Brown P Botstein D Cluster analysis and display of genome-wide expression patterns PNAS 1998 95 25 14863 68 10.1073/pnas.95.25.14863 9843981 PMC24541

[B21] Gasch A Spellman P Kao C Carmel-Harel O Eisen Mea Genomic expression programs in the response of yeast cells to environmental changes Mol Biol Cell 2000 11 12 4241 57 11102521 10.1091/mbc.11.12.4241 PMC15070

[B22] Tavazoie S Hughes J Campbell M Cho R Church G Systematic determination of genetic network architecture Nat Genet 1999 22 3 281 85 10.1038/10343 10391217

[B23] Ji L Tan KL Identifying time-lagged gene clusters using gene expression data Bioinformatics 2005 21 4 509 516 10.1093/bioinformatics/bti026 15374868

[B24] Chen T Filkov V Skiena S (Eds) Identifying gene regulatory networks from experimental data 1999

[B25] Kwon A Hoos H Ng R Inference of transcriptional regulation relationships from gene expression data Bioinformatics 2003 19 905 912 10.1093/bioinformatics/btg106 12761051

[B26] Balasubramaniyan R Hullermeier E Weskamp N Kamper J Clustering of gene expression data using a local shape-based similarity measure Bioinformatics 2005 21 7 1069 77 10.1093/bioinformatics/bti095 15513997

[B27] Ernst J Bar-Joseph Z STEM: a tool for the analysis of short time series gene expression data BMC Bioinformatics 2006 7 1 191 10.1186/1471-2105-7-191 16597342 PMC1456994

[B28] Yeung L Szeto L Liew A Yan H Dominant spectral component analysis for transcriptional regulations using microarray time-series data Bioinformatics 2004 20 742 749 10.1093/bioinformatics/btg479 14751991

[B29] Ng A Jordan M Weiss Y On spectral clustering: Analysis and an algorithm Advances in Neural Information Processing Systems 2002 14

[B30] Gower JC Ross GJ Minimum spanning trees and single linkage analysis Appl Stat 1969 18 54 64 10.2307/2346439

[B31] Xu Y Olman V Xu D Clustering gene expression data using a graphtheoretic approach: an application of minimum spanning trees Bioinformatics 2002 18 4 536 45 10.1093/bioinformatics/18.4.536 12016051

[B32] McLachlan GJ Bean RW Peel D A mixture model-based approach to the clustering of microarray expression data Bioinformatics 2002 18 413 422 10.1093/bioinformatics/18.3.413 11934740

[B33] Ng S McLachlan GJ Wang K Jones LBT Ng SW A mixture model with random-effects components for clustering correlated gene-expression profiles Bioinformatics 2006 22 1745 1752 10.1093/bioinformatics/btl165 16675467

[B34] Yuan Y Li CT Wilson R Partial mixture model for tight clustering of gene expression time-course BMC Bioinformatics 2008 9 287 10.1186/1471-2105-9-287 18564420 PMC2492882

[B35] Pan W Lin J Le CT Model-based cluster analysis of microarray geneexpression data Genome Biol 2002 3 2 RESEARCH0009 10.1186/gb-2002-3-2-research0009 11864371 PMC65687

[B36] Dempster A Laird N Rubin D Maximum likelihood from incomplete data via the EM algorithm J Royal Stat Soc 1977 B-39 1 38

[B37] Schliep A Schonhuth A Steinhoff C Using hidden Markov models to analyze gene expression time course data Bioinformatics 2003 19 264 272 10.1093/bioinformatics/btg1036 12855468

[B38] Schliep A Costa I Steinhoff C Schonhuth A Analyzing Gene Expression Time-Courses IEEE/ACM Transactions on computational biology and bioinformatics 2005 2 3 179 193 10.1109/TCBB.2005.31 17044182

[B39] Ramoni PMF Sebastiani Kohane I Cluster analysis of gene expression dynamics PNAS 2002 99 9121 9126 10.1073/pnas.132656399 12082179 PMC123104

[B40] Bar-Joseph Z Gerber G Jaakkola T Gifford D Simon I Continuous representations of time series gene expression data J Comput Biol 2003 3 4 341 356 10.1089/10665270360688057 12935332

[B41] Zhao L Prentice R Breeden L Statistical modeling of large microarray data sets to identify stimulus response profiles PNAS 2001 98 5631 5636 10.1073/pnas.101013198 11344303 PMC33264

[B42] Lu X Zhang W Qin Z Kwast K Liu J Statistical resynchronization and Bayesian detection of periodically expressed genes Nucleic Acids Res 2004 32 447 455 10.1093/nar/gkh205 14739237 PMC373332

[B43] Moller-Levet C Chu K Wolkenhauer O DNA microarray data clustering based on temporal variation: Fcv with tsd preclustering Appl Bioinformatics 2003 2 35 45 15130832

[B44] Lim PO Kim Y Breeze E Koo JC Woo HR Ryu JS Park DH Beynon J Tabrett A Buchanan-Wollaston V Nam HG Overexpression of a chromatin architecture-controlling AT-hook protein extends leaf longevity and increases the post-harvest storage life of plants The Plant Journal 2007 52 1140 1153 17971039 10.1111/j.1365-313X.2007.03317.x

[B45] Gene Ontology: tool for the unification of biology Nature Genet 2000 25 25 29 10.1038/75556 10802651 PMC3037419

[B46] Maere S Heymans K Kuiper M BiNGO: a Cytoscape plugin to assess overrepresentation of Gene Ontology categories in biological networks Bioinformatics 2005 21 3448 3449 10.1093/bioinformatics/bti551 15972284

[B47] Benjamini Y Hochberg Y Controlling the false discovery rate: A practical and powerful approach to multiple testing J R Stat Soc 1995 B 57 289 300

[B48] Feng JF Yi D Krishna R Guo S Buchanan-Wollaston V Listen to Genes: Dealing with Microarray Data in the Frequency Domain PLos ONE 2009 4 4 e5098 10.1371/journal.pone.0005098 PMC3383793 22745650

[B49] Barabsi AL Albert R Emergence of scaling in random networks Science 1999 286 509 512 10.1126/science.286.5439.509 10521342

[B50] Watts DJ Strogatz SH Collective dynamics of 'small-world' networks Nature 1998 393 440 442 10.1038/30918 9623998

[B51] Goldberg DS Roth FP Assessing experimentally derived interactions in a small world PNAS 2003 100 4372 4376 10.1073/pnas.0735871100 12676999 PMC404686

[B52] Ravasz E Somera AL Mongru DA Oltvai ZN Barabasi AL Hierarchical organization of modularity in metabolic networks Science 2002 297 1551 1555 10.1126/science.1073374 12202830

[B53] Angelini C Cutillo L De Canditiis D Mutarelli M Pensky M BATS: a Bayesian user-friendly software for Analyzing Time Series microarray experiments BMC Bioinformatics 2008 9 1 415 10.1186/1471-2105-9-415 18837969 PMC2579305

[B54] Ancona N Marinazzo D Stramaglia S Radial basis function approach to nonlinear Granger causality of time series Physical Review E 2004 70 056221 10.1103/PhysRevE.70.056221 15600742

[B55] Marinazzo D Pellicoro M Stramaglia S Nonlinear parametric model for Granger causality of time series Physical Review E 2006 73 066216 10.1103/PhysRevE.73.066216 16906955

[B56] Pihur V Datta S Datta S Reconstruction of genetic association networks from microarray data: a partial least squares approach Bioinformatics 2008 24 4 561 568 10.1093/bioinformatics/btm640 18204062

[B57] Schafer J Strimmer K An empirical Bayes approach to inferring large-scale gene association networks Bioinformatics 2005 21 6 754 764 10.1093/bioinformatics/bti062 15479708

[B58] Granger C Newbold P Forecasting Economic Time Series 1986 Academic Press

[B59] Schwert GW Tests of causality: The message in the innovations Carnegie-Rochester Conference Series on Public Policy 1979 10 1 55 96 10.1016/0167-2231(79)90003-4

[B60] Akaike H Fitting autoregressive models for regression Annals of the Institute of Statistical Mathematics 1969 21 243 247 10.1007/BF02532251

[B61] Shannon P Markiel A Ozier O Baliga NS Wang JT Ramage D Amin N Schwikowski B Ideker T Cytoscape: a software environment for integrated models of biomolecular interaction networks Genome Research 2003 13 11 2498 504 10.1101/gr.1239303 14597658 PMC403769

[B62] Xenarios I Salwinski L Duan XJ Higney P Kim SM Eisenberg D DIP, the Database of Interacting Proteins: a research tool for studying cellular networks of protein interactions Nucleic Acids Res 2002 30 303 305 10.1093/nar/30.1.303 11752321 PMC99070

[B63] Dehmer M Emmert-Streib F (Eds) Analysis of Microarray Data: A Network-Based Approach 2008 Wiley-VCH

[B64] Goldberg A Finding a Maximum Density Subgraph 1984 Tech rep, EECS Department, University of California, Berkeley

[B65] Bader G Hogue C An automated method for finding molecular complexes in large protein interaction networks BMC Bioinformatics 2003 4 2 10.1186/1471-2105-4-2 PMC149346 12525261

